# Role of FlhF and its domains in the assembly of a polar flagellum in *P. aeruginosa*

**DOI:** 10.1128/jb.00332-25

**Published:** 2025-11-24

**Authors:** Shikha Raghav, Rohit Prajapati, Deepti Jain

**Affiliations:** 1 Transcription Regulation Lab, Regional Centre for Biotechnology, NCR Biotech Science Cluster 682813 , Faridabad, India; Geisel School of Medicine at Dartmouth, Hanover, New Hampshire, USA

**Keywords:** FlhF, *Pseudomonas aeruginosa*, FliG, flagella, polarity

## Abstract

FlhF, an SRP GTPase, regulates the polarity and flagellation patterns in various bacteria, including *Pseudomonas aeruginosa*. FlhF is a multidomain protein that comprises three domains: B, N, and G, or the GTPase domain. However, the roles of different domains in the assembly and maintenance of flagella in this clinically important bacterium remain unclear. In this study, we report that the C-terminal GTPase domain plays a critical role in maintaining the polar localization of FlhF. Complementation assays using truncated constructs show that the flagellar assembly depends on both the B- and N-domains. In addition, GTP binding by FlhF regulates its interaction with FliG, a flagellar C-ring protein that facilitates flagellar basal body assembly in *P. aeruginosa*.

**IMPORTANCE:**

Pathogenic bacteria rely on flagellar motility for infection and colonization. FlhF, a member of the SRP GTPase family, is a key factor regulating flagellation patterns in various bacteria. In this study, we investigated the domain-wise role of FlhF in *P. aeruginosa,* an *ESKAPE* pathogen that uses a single flagellum for its virulence, biofilm formation, and pathogenesis. Our study reveals the role of FlhF in the assembly and spatial regulation of flagella in *P. aeruginosa*. In addition, we examined the interaction of FlhF with the flagellar ring protein. This study provides insights into the molecular mechanism underlying polar assembly of flagella, thus offering a targeted approach for controlling infection by this pathogen.

## INTRODUCTION

Flagella serve as the organ of motility in bacteria, supporting survival and adaptation to myriad environmental conditions. Although the function and structural organization of flagella are conserved across bacterial species, their number and placement are species-specific. The flagellation pattern is tightly regulated and maintained over generations (1). *Pseudomonas aeruginosa* is an opportunistic human pathogen that possesses a single polar flagellum (2, 3). The presence of flagella in these bacteria is vital for their virulence, pathogenesis, and transition from the flagellated to the biofilm mode of life. Furthermore, it has been shown that the aflagellated cells fail to form robust biofilms (4–6). Thus, a comprehensive study of the factors involved in establishing the spatiotemporal regulation of flagella is imperative.

Flagellar gene expression in *P. aeruginosa* is tightly regulated by a multilayered, ordered transcription cascade. FleQ, a class I master transcription factor, sits at the top of the regulatory hierarchy and controls the expression of class II genes in association with σ^54^ (7). These genes encode proteins that make up the basal body, motor and export apparatus, and a few regulatory proteins. The activity of FleQ is, in turn, regulated through interaction with a Class II protein, FleN (8–10). The expression of class III genes, which include hook proteins, is controlled by a two-component system, FleSR (11). Class IV genes, including *fliC*, are transcribed by σ^28^ (12).

FlhF is a 48 kDa class II flagellar polarity regulator that belongs to the SIMIBI class of nucleotide triphosphate-binding proteins. Along with two other members of this class, the signal recognition particle (SRP) and SRP receptor (FtsY), these GTPases form a subset within this class of proteins (13, 14). In polar monoflagellates, such as *Pseudomonas*, *Vibrio,* and *Shewanella,* FlhF is the key protein required for flagellar biogenesis and localizes to the bacterial cell poles (15–20). FlhF knockouts either lack flagella or exhibit mislocalization at non-polar sites with defective swimming motility in different bacterial species (21–28).

FlhF has a modular architecture comprising an N-terminal B-domain, central N-domain, and C-terminal GTPase domain. The crystal structure of FlhF from *Bacillus subtilis* shows that it comprises GTP-bound NG-domains arranged as a homodimer with each monomer contributing to the formation of the composite active site. Unlike in the case of Ffh and FtsY, which form a heterodimer mediated by N- and G-domains, the dimer interface of FlhF is present only in the GTPase domain (13, 29, 30). Sequence alignment of FlhF from different bacterial species has revealed that the B- and N-domains are highly variable, whereas the GTPase domain is conserved and harbors motifs necessary for GTP binding and hydrolysis (G1-G5).

The B-domain is often described as “basic” because of its amino acid composition. It is predicted to be largely disordered, with a small structured domain at the N-terminus . In *Shewanella*, this domain has been shown to interact with FliG, a key component of the flagellar C-ring (31). In *Vibrio cholerae*, both the B-domain and the GTP-binding region are critical for recruiting FliF—a key component of the MS-ring—to the cell pole, while the central N-domain plays a role in polar localization of the FlhF (32). In *C. jejuni,* the GTP-binding site mutants show reduced motility and flagellation at the lateral position (25). In *S. oneidensis,* GTPase activity is required for motility but does not affect the position of the flagella (16). Furthermore, GTP binding, but not hydrolysis, is required for flagellar localization in *P. aeruginosa* and *Vibrio alginolyticus* (15, 33). FlhF, through its NG-domain, interacts with HubP, a polar landmark protein in *S. putrefaciens,* in a GTP-independent manner (31).

These studies emphasize that the functions of individual domains of FlhF may differ among bacterial species. Therefore, investigations on this clinically important bacterium are warranted. In this study, we functionally characterized various domains of FlhF from *P. aeruginosa* PAO1, providing evidence that all three domains of FlhF are essential for bacterial motility. We show that B- and N-domains are required for flagellar assembly, and the GTPase domain of the protein is crucial for the polar flagellar phenotype. We also demonstrate that FlhF, when bound to GTP, interacts with the C-ring protein FliG and supports flagellar assembly in *P. aeruginosa*.

## RESULTS

### All domains of FlhF are necessary for swimming motility in *P. aeruginosa*

**Fig 1:**
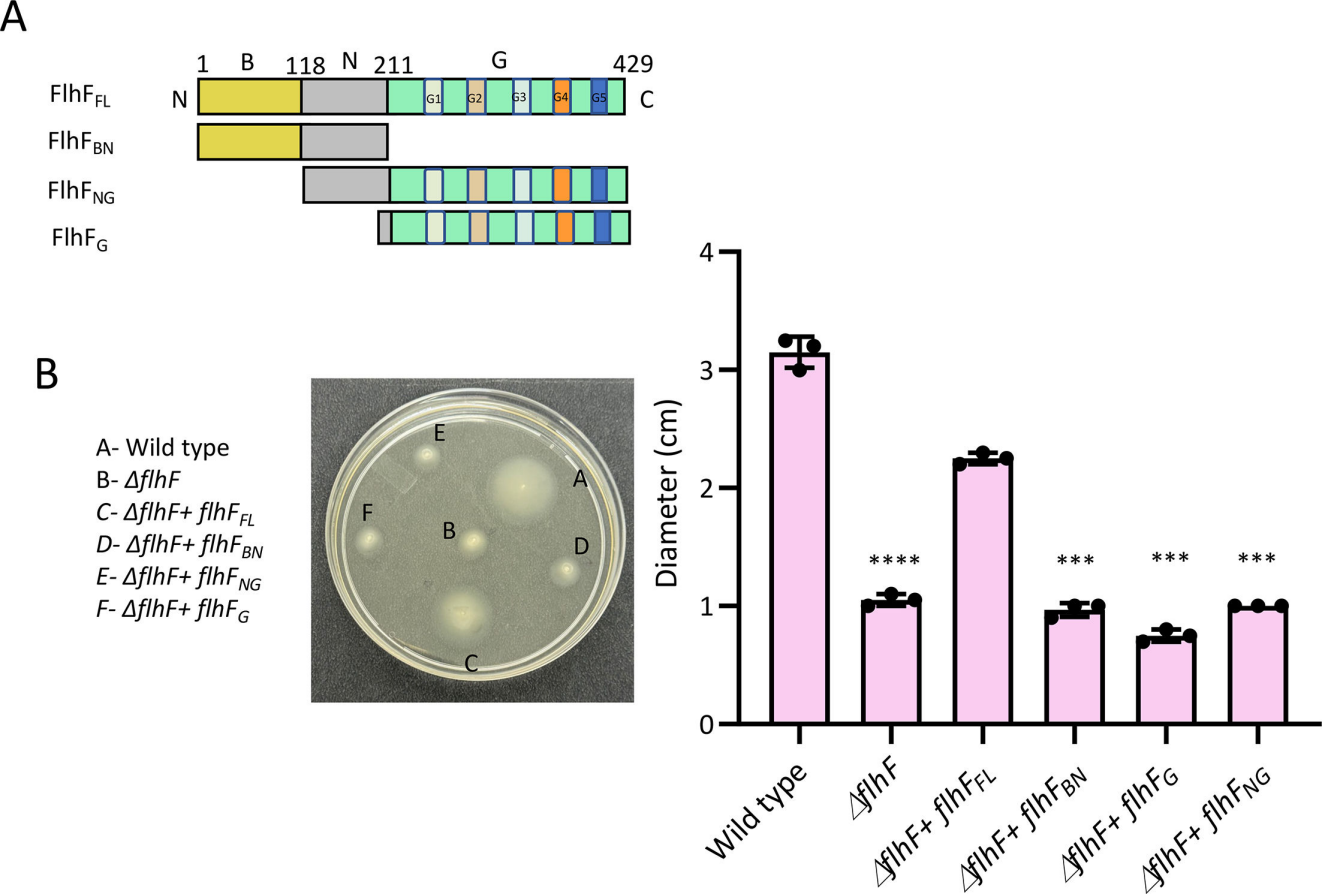
(**A**) Domain organization of FlhF from *P. aeruginosa*. The schematic shows the amino acids corresponding to each domain. The domains are labeled as B, N, and G. The conserved motifs of the GTPase domain are represented as colored rectangles and labeled as G1, G2, G3, G4, and G5. (**B**) Image of soft agar plate depicting swimming motility of the wild-type PAO1, *flhF* knockout (*∆flhF*), knockout complemented with *flhF*_FL_, *flhF_BN_*, *flhF*_NG_, or *flhF*_G_ domains. The bar graph represents the average diameter from the three independent experiments. The significance was determined using an unpaired t-test. The significance of the knockout of *flhF* was calculated with respect to wild type, whereas for *ΔflhF + flhF_BN_, ΔflhF + flhF_NG_* and *ΔflhF + flhF_G_*, the comparison was made with respect to *ΔflhF + flhF_FL_*. The statistical significance when compared with respective controls is indicated as *P < 0.05, **P < 0.01, and ***P < 0.001.

We first prepared a series of gene constructs of *flhF* to probe the roles of various domains. The *flhF_BN_* (corresponding to amino acid residues 1–211), the *flhF_NG_* (118–429), and the *flhF_G_* (202–429) domains were PCR amplified and cloned into pHERD20T, an arabinose-inducible vector containing a *pBAD* promoter for regulated gene expression in *Pseudomonas* (34). To investigate the role of different domains of FlhF in maintaining optimal motility of *P. aeruginosa* PAO1, a swimming motility assay was performed. The diameter of the bacterial spread on soft agar plates representing the motility of the strains was measured. While the wild-type PAO1 strain displayed the largest motility zone (3.1 ± 0.2 cm), the *flhF_FL_* knockout showed impaired motility with a diameter of 1 ± 0.1 cm. The motility of the complemented strains with construct containing either the full-length FlhF (*flhF*_FL_) or any of the domains *flhF*_BN_ *flhF*_NG_, or *flhF_G_* was also evaluated (Fig. 1A). Complementation of the *flhF_FL_* restored motility by up to 2.2 ± 0.1 cm. Surprisingly, deletion of either of the domains, B, N, or G, failed to reinstate motility and led to a reduction of about 67% compared to the strain complemented with *flhF_FL_*. This suggests that the presence of all domains of FlhF is required for ensuring optimal motility in *P. aeruginosa* (Fig. 1B). Complementation of these constructs in the wild-type bacteria was also evaluated, which resulted in motility comparable to that of the PAO1 strain (Fig. S1).

### The FlhF_B_ domain is vital for flagellar assembly

**Fig 2:**
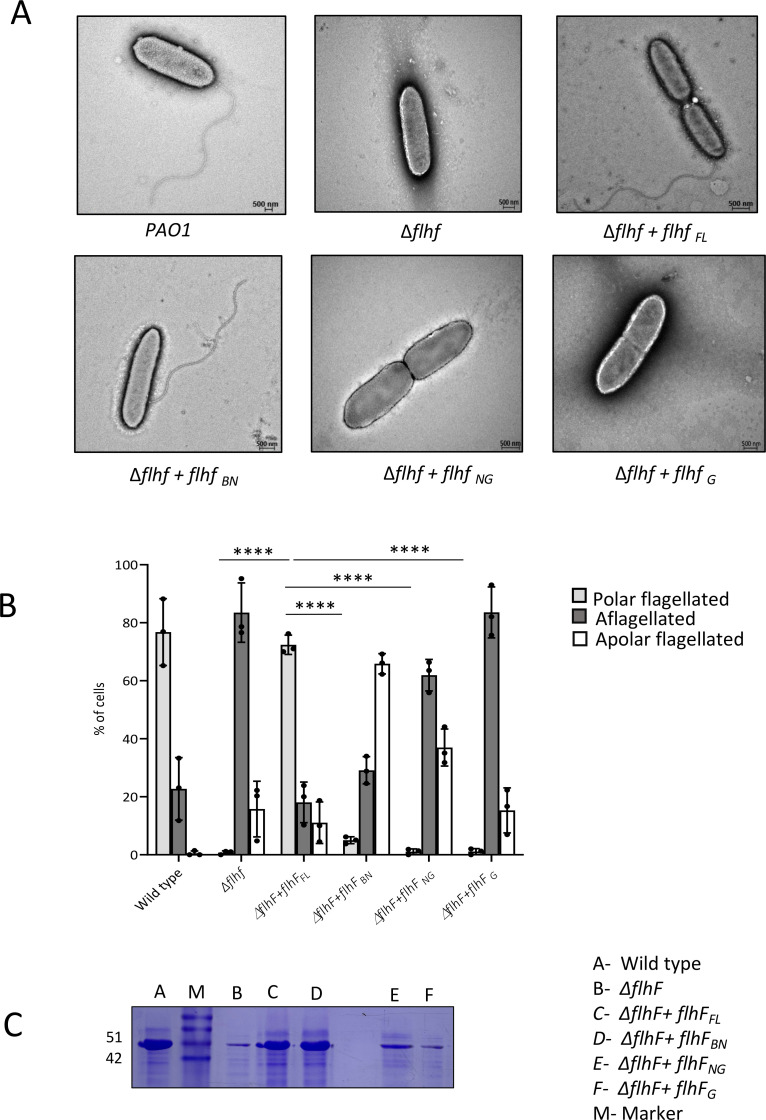
(**A**) The upper panel shows electron micrographs depicting the flagellar phenotypes of the wild-type PAO1, *∆flhF*, and *∆flhF* complemented with *flhF_FL_*. The lower panel shows the flagellar phenotypes of the *∆flhF* strain complemented with *flhF*_BN_, *flhF*_NG_, and *flhF*_G_. (**B**) The data presented in the bar graph show the percentage of polar flagellated, aflagellated, and non-polar flagellated bacterial cells. A total of 100 cells were counted for each experiment, and three independent experiments were performed. The error bars represent the standard deviation. The significance was determined using an unpaired t-test. The statistical significance when compared with respective controls is indicated as follows: *P < 0.05, **P < 0.01, and ***P < 0.001. (**C**) Picture of Coomassie-stained 10% SDS-PAGE gel for isolated flagellins from different strains as mentioned.

The single polar flagellum of *P. aeruginosa* is a prerequisite for swimming and swarming motility (35). Since deletion of individual domains led to severely compromised motility, transmission electron microscopy (TEM) was performed on the *P. aeruginosa* wild type*, ∆flhF,* and strains complemented with different domain constructs to investigate the flagellar phenotypes. 83% ± 9% of the cells lacking *flhF_FL_* were aflagellate but exhibited a single polar flagellum similar to the wild type, once *flhF_FL_* was ectopically expressed on the plasmid pHERD20T (Fig. 2). However, complementation of the knockout strain with constructs comprising either the *flhF_NG_* or *flhF_G_* resulted in the absence of flagella. Surprisingly, when the *flhF_BN_* was complemented, approximately 65% ± 5% of the cells showed a mislocalized, non-polar flagellum compared to a polar flagellum in 73% ± 4% of cells complemented with *flhF*_FL_ (Fig. 2). Hence, the results suggest that although the B-domain supports flagella formation, the presence of the GTPase domain is obligatory to restrict flagella development at the poles in *P. aeruginosa*.

### The GTPase domain aids in the polar localization of FlhF

**Fig 3:**
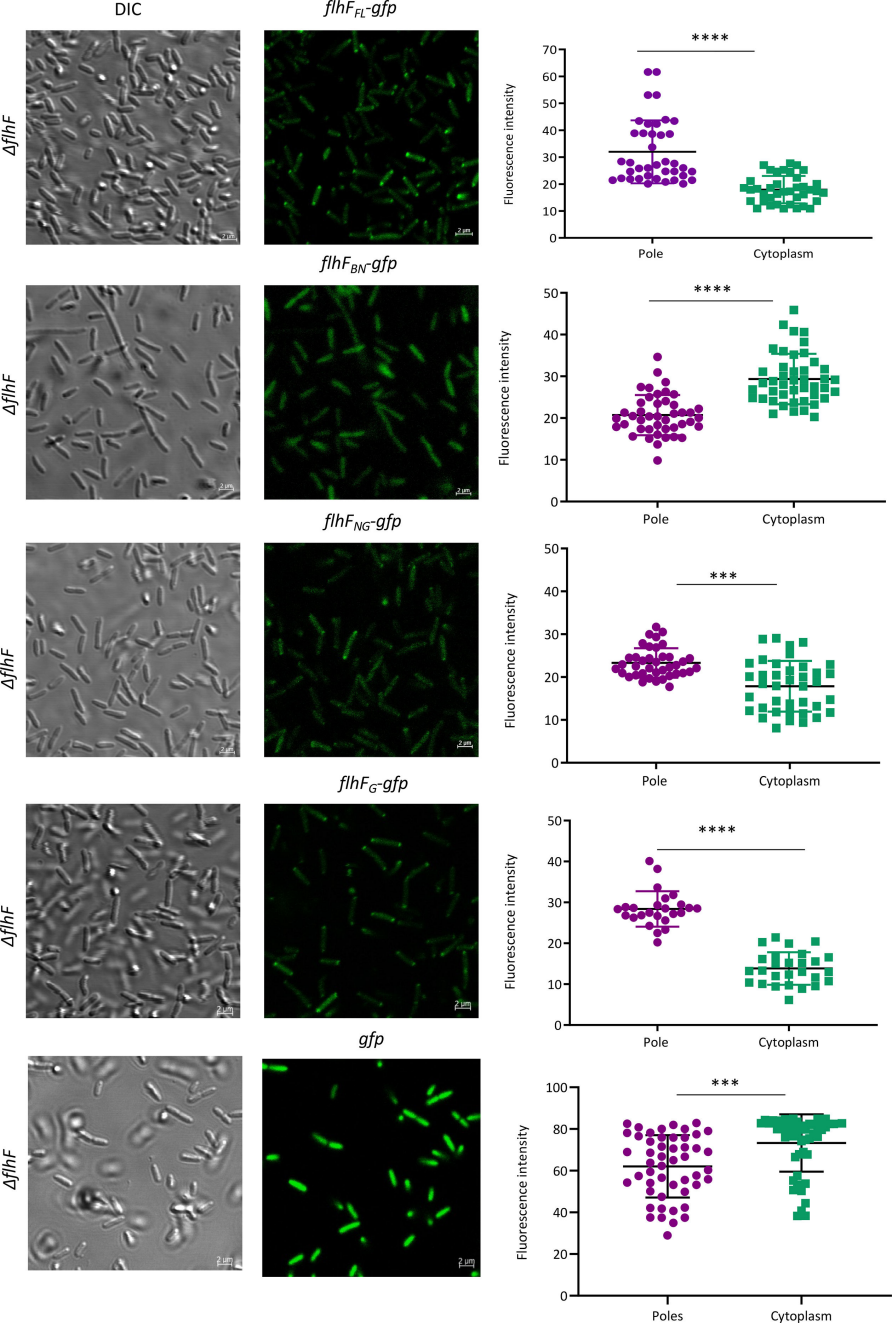
Protein localization. Differential interference contrast (DIC) and fluorescence microscopy images of *∆flhF* strains complemented with *flhF_FL_-gfp, flhF_BN_-gfp, flhF_NG_-gfp, flhF_G_-gfp,* and *gfp* are shown. The right side of each panel shows a dot plot, where each dot represents intensity measured at the pole and cytoplasm of a single bacterial cell. The plot shows results from three independent experiments. Intensities were measured using ImageJ, and a dot plot was plotted using GraphPad Prism. The mean value and standard deviation were included in the dot plot. The statistical significance is indicated as follows: ****P < 0.0001, *P < 0.05, **P < 0.01, and ***P < 0.001.

Our results suggest that FlhF is a key determinant of motility and flagellar polarity in *P. aeruginosa*. Thus, the influence of various domains on the intracellular localization of the GFP-tagged protein was examined. To determine the localization of FlhF and its domains, we used a C-terminal-GFP fusion protein for complementation and performed confocal microscopy to visualize GFP-tagged FlhF_FL_ inside the bacterial cells. The expression of the constructs was verified using Western blot (Fig. S2). We observed that *flhf_FL_-gfp* complemented *∆flhF* cells had protein localized as bright spots at the poles (Fig. 3). However, when knockouts were complemented with a pHERD20T vector containing *gfp* as a control, the fluorescence was diffused throughout the cytoplasm (Fig. 3). Similarly, polar localization for the proteins was observed when the *flhF_G_* or *flhF_NG_* domains tagged with GFP were complemented into *∆flhF* cells. Remarkably, complementation with the construct lacking the G-domain (containing only *flhF_BN_*) resulted in a defective polar localization, and the protein was primarily diffused in the cytoplasm (Fig. 3). These observations imply that the G-domain predominantly governs the localization of FlhF_FL_ at the poles, as its absence disrupts the polar positioning of FlhF, consequently affecting the position of the flagella in bacterial cells. Another interesting observation is that the accumulation of the GTPase domain at the poles in the absence of the B-domain does not support flagellation in *P. aeruginosa,* as evident from TEM experiment, which implies that the B-domain plays a role in flagellar biogenesis, whereas the GTPase domain of FlhF maintains the polarity of the protein and hence basal body assembly site.

### FlhF interacts with the C-ring protein FliG

The *in vivo* complementation assays presented above reveal that FlhF is indispensable for the formation of polar flagella. This GTPase supports the assembly of the basal body in different species of bacteria by recruiting flagellar ring proteins. The C-ring lies on the cytoplasmic side of the flagellar basal body. This ring acts as a rotor to regulate the directional switching (36, 37). To elucidate the molecular mechanism of polar flagellum assembly in *P. aeruginosa*, we hypothesized that FlhF interacts with FliG, a component of the C-ring that interfaces with the MS-ring (31, 38).

**Fig 4:**
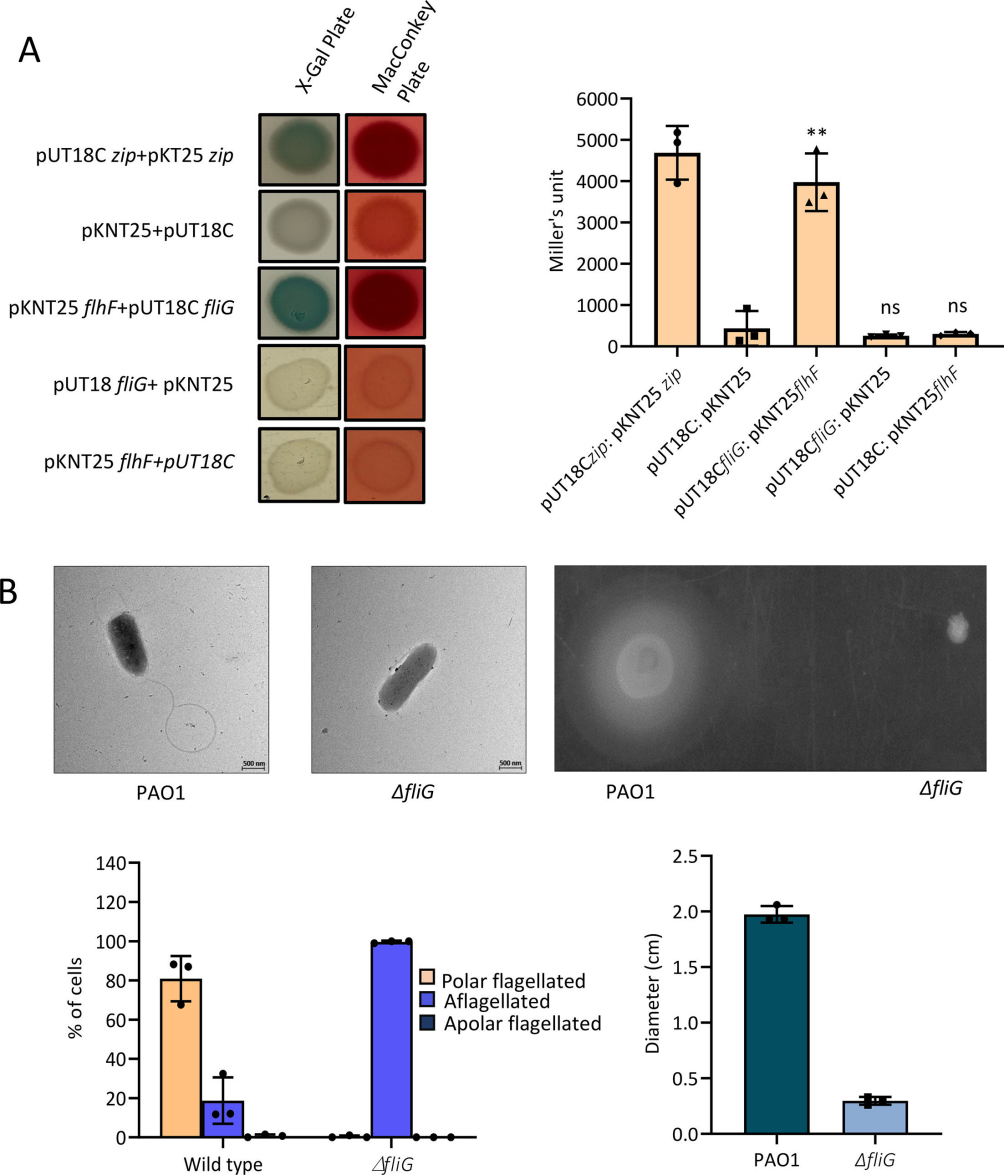
(**A**) Interaction of FlhF with the C-ring protein FliG. Bacterial two-hybrid assay depicting interaction between FlhF and FliG. BTH101 cells co-transformed with sets of plasmids containing *flhF* and *fliG* were plated on LB agar plates with X-gal and IPTG on the left and on MacConkey indicator plate with maltose on the right, along with positive and negative controls. The bar graph represents the quantification of β-galactosidase activity in Miller’s units. The measure of activity gives the readout for functional complementation brought about by the interaction of the two proteins, leading to cya + phenotypes. The statistical significance when compared with cells co-transformed with empty vectors is indicated as follows: **P < 0.05; ns, non-significant. (**B**) Electron micrographs showing the flagellar phenotypes of the wild-type PAO1 and *fliG* knockout are shown along with their motility on soft agar plates. The bar graphs were prepared using data from three independent experiments.

We next probed the interaction between FlhF and FliG, using an adenylate cyclase-based bacterial two-hybrid assay (BATCH). The *flhF*, cloned in pKNT25 (containing T25 fragment fused at the downstream of the cloned gene), was shown to interact strongly with pUT18C-*fliG* (containing T18 fragment upstream of the cloned gene) in BACTH. The co-transformants produced strong pink and bluish-green color colonies on MacConkey and X-gal plates, respectively, indicating high β-galactosidase activity. These observations suggest an interaction between FlhF and FliG, consistent with findings from a previous study (Fig. 4A) (39). However, another key inference from this experiment was that the interaction between the two proteins occurred when the N- and C-terminal domains of FlhF and FliG, respectively, were freely accessible (Fig. 4A).

We also evaluated the flagellar phenotype and swimming motility of the Δ*fliG* strain of *P. aeruginosa* using TEM and soft agar assay, respectively. There was a loss of flagella in 100% of the cell population upon knockout of *fliG*. The cells also showed defective swimming motility, as measured by the spread of the zone on the soft agar plates (Fig. 4B). FliG is a component of the C-ring, and the absence of flagella in Δ*fliG* suggests that it is indispensable for flagella formation in *P. aeruginosa*.

### FlhF and FliG interact in the presence of GTP analog

**Fig 5:**
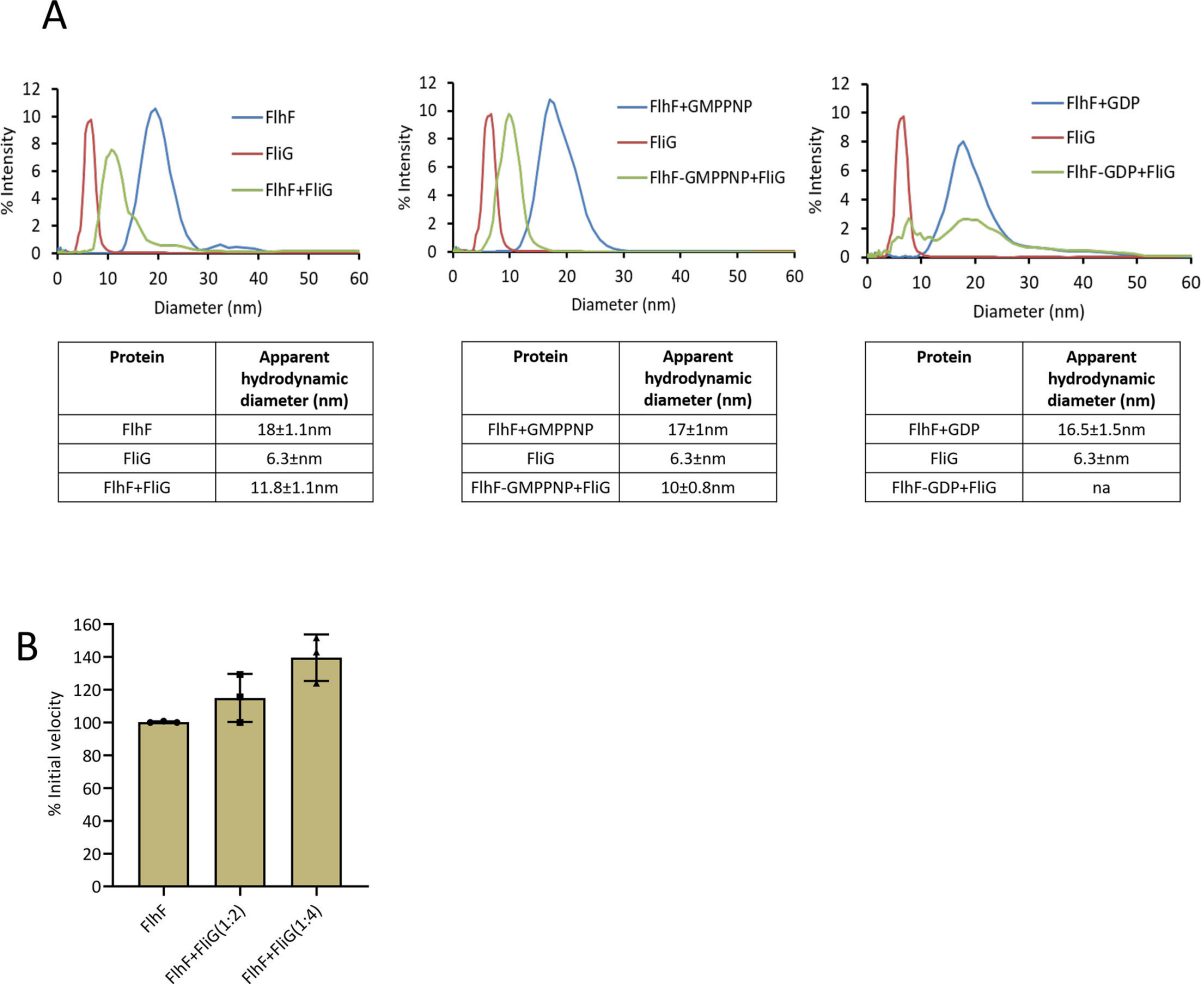
Interaction of FlhF with the C-ring protein FliG in the presence of nucleotides. (**A**) Distribution of the hydrodynamic diameters for FlhF (blue), FliG (red), and the complex (green) obtained by DLS is plotted as a function of intensity. The second and third panels represent the distribution of intensities when complex formation was tested in the presence of GMPPNP and GDP, respectively. The table below each panel shows the average hydrodynamic diameter with standard deviations. (**B**) The GTPase activity of FlhF was measured in the presence of FliG at 1:2 and 1:4 molar ratios. The graph represents the average of three independent replicates with standard deviations.

To further characterize the interaction between the proteins, we cloned, expressed, and purified full-length FlhF and FliG and performed dynamic light scattering (DLS) experiments to evaluate their binding. A picture of SDS-PAGE gel showing purified proteins is depicted in Fig. S3. The hydrodynamic diameter of full-length FlhF was found to be close to 18 nm, while that of free FliG was around 6 nm. The complex formed with a 1:1 molar ratio of the two proteins showed a diameter between those of the two proteins, as depicted in Fig. 5A. Similarly, when FliG was allowed to form a complex with FlhF bound to a non-hydrolysable analog of GTP, Guanosine-5′-[(β, γ)-imido]triphosphate (GMPPNP), a sharp, monodisperse peak was observed for complex formation, as shown in Fig. 5A. Interestingly, the interaction between the two proteins was disturbed when FlhF was preincubated with guanosine diphosphate (GDP), suggesting that GDP binding to FlhF prevents its interaction with FliG (Fig. 5A). Overall, the data indicate that FlhF interacts with FliG in the GTP-bound state and that this interaction is unstable in the presence of GDP. We hypothesize that FlhF recruits FliG to the bacterial poles, and hydrolysis of GTP to GDP weakens the FlhF–FliG complex, causing the release of FlhF.

GTPase activity is regulated by GTPase-activating proteins and guanine exchange factors (40). Next, we examined whether the GTPase activity of FlhF was influenced by increasing concentrations of FliG. No stimulation of GTPase activity was observed when FliG was added at a twofold molar ratio of FlhF, whereas the GTPase activity of FlhF showed a marginal increment when a fourfold molar ratio of FliG was used (Fig. 5B). Thus, we conclude that the GTPase activity of FlhF is not stimulated in the presence of FliG. This is also consistent with the DLS data, which show that GTP binding is important for stable complex formation between FlhF and FliG.

## DISCUSSION

*P. aeruginosa* is a monoflagellated, clinically important bacterium. It is the leading cause of nosocomial infections in immunocompromised individuals (41). It has a single polar flagellum that aids in motility and biofilm formation (4, 42). FlhF, a protein belonging to the SRP GTPase family, plays a crucial role in maintaining flagellation patterns in different bacterial species (1, 43). However, to date, no study has systematically investigated the roles of the B-, N-, and G-domains in *P. aeruginosa*. We therefore conducted *in vivo* analyses using truncated variants of FlhF to elucidate the function of each domain.

FlhF is a class II protein in the transcriptional hierarchy of flagellar genes in *P. aeruginosa* (44). In this study, we showed that FlhF is essential for flagellar assembly and its polar localization. Specifically, we demonstrate that the G-domain or GTPase domain directs the FlhF to the bacterial cell pole. In the absence of this domain, FlhF is dispersed throughout the cytoplasm, impairing the polar placement of flagella. It has been reported that the localization of FlhF is independent of other flagellar components, although transmembrane proteins, such as HubP, FipA, and the membrane-associated TonB3-PocA-PocB complex, contribute to maintaining FlhF at the poles (19, 45, 46). The spatiotemporal distribution of these proteins needs to be determined to decipher the exact molecular mechanism supporting the terminal localization of FlhF.

We also demonstrate that the FlhF B-domain, irrespective of its distribution in the cytoplasm or poles, is indispensable for flagellar assembly, likely serving as a mediator connecting FlhF G-domain and basal body components. The observations made in our study were consistent with the findings observed in *S. putrefaciens* (16). The importance of the B-domain is underscored by the fact that its absence results in non-flagellated cells despite proper localization of FlhF, emphasizing its role in the initiation of flagella synthesis. The flagellar basal body of gram-negative bacteria comprises four different rings (47). FlhF aids in the assembly of the MS-ring, present in the inner membrane (48). This ring is made of 34 molecules of the protein FliF, each composed of two transmembrane and a periplasmic region with C- and N-termini in the cytoplasmic region. The N-terminus of FliF was shown to interact with FlhF, and this interaction was critical for the assembly of the MS-ring in *V. alginolyticus*. However, in the case of *Salmonella,* FliF alone was sufficient for assembling the MS-ring (49). The C-terminus region of FliF, on the other hand, has been shown to interact with the N-terminus of FliG, which is one of the constituents of the C-ring in *E. coli*, *Salmonella*, and *T. maritima* (50–53). Sequence alignment of FliG from various polarly flagellated bacteria shows a high degree of sequence conservation among all three domains: N-terminal, middle, and C-terminal, indicating the possibility of conservation of this interaction (54).

We also report protein–protein interaction between FlhF and C-ring protein, FliG, *in vivo* using bacterial two-hybrid assay. It was observed that in the absence of either of the two proteins, in the bacterial cells, the flagella failed to assemble, highlighting the importance of this interaction. This interaction occurred only when the N-terminus of FlhF was freely available, indicating the possibility of interaction with the B-domain. The C-ring assembles on the cytoplasmic surface of the cell and also contains the proteins FliM and FliN, which do not interact with FlhF (31). The FlhF-FliG interaction was seen in the polar flagellated system and was absent in the lateral flagellar system of *S. putrefaciens*. Although the protein–protein interface is not yet defined, the N-terminal 60 amino acids of the B-domain of FlhF and the middle and C-terminal domains of FliG are likely involved (31). *P. aeruginosa* shows 50% and 62% sequence identity with *S. putrefaciens* in these respective domains, indicating a similar interface.

**Fig 6:**
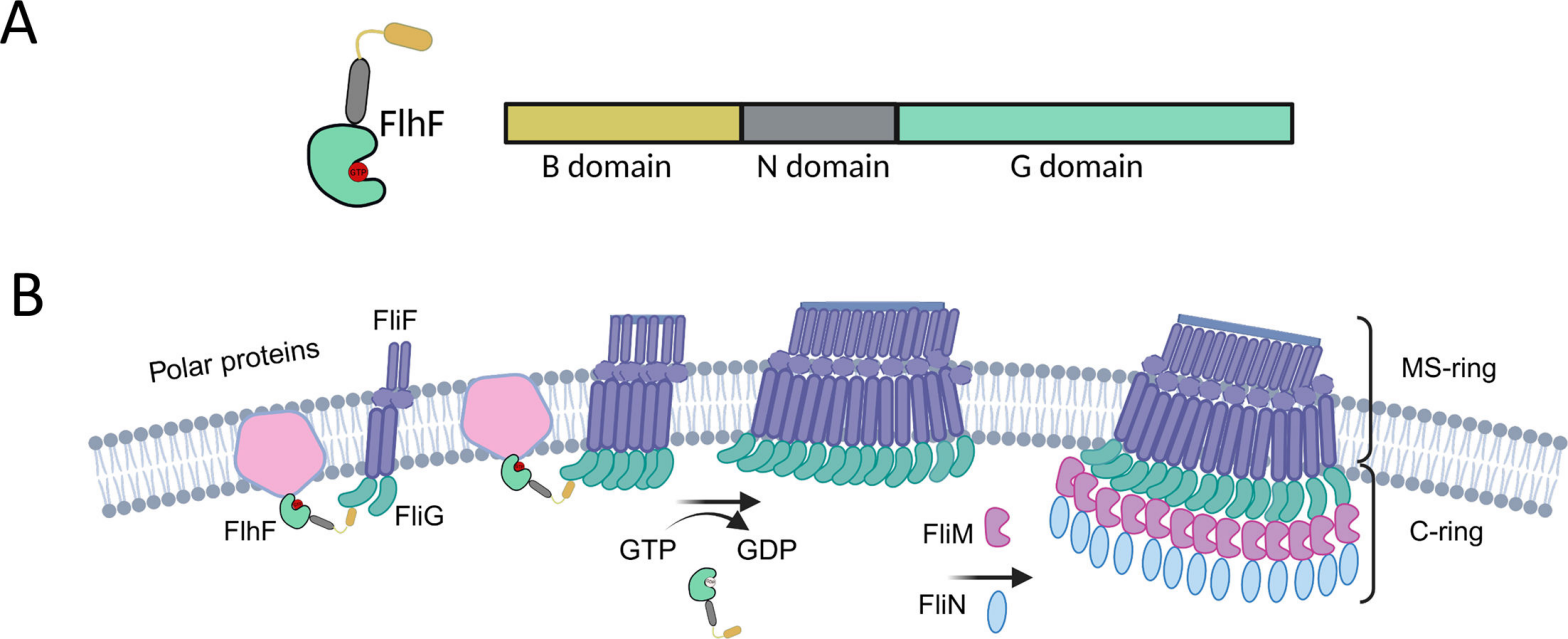
Schematic representation of the model for basal body assembly in *P. aeruginosa*. (**A**) Domain organization of FlhF along with a schematic of the protein showing B (yellow), N (gray), and G (green) domains. (**B**) Proposed mechanism of flagellar assembly where FlhF localizes at the poles via its GTPase domain, possibly through interaction with polar protein (pink). In the presence of bound GTP, it interacts with FliG (aquamarine-green) and recruits it at the pole, facilitating its interaction with FliF (light purple). Post-GTP hydrolysis, FlhF dissociates, leaving FliG free to interact with FliM (magenta) and FliN (blue), resulting in the assembly of the C-ring.

We further found that GTP-bound FlhF interacts with FliG, and upon hydrolysis, the interaction between the two proteins is weakened, and perhaps FlhF is released from the complex in *P. aeruginosa*. However, the FlhF-FliG interaction prevents FliG from interacting with FliM and FliN. Thus, we speculate that the release of FlhF from the FlhF-FliG complex upon GTP hydrolysis makes FliG available to interact with other components of the C-ring (Fig. 6). While closely examining the role of each domain of FliG, it was apparent that the middle and C-terminal domains contain residues important for interaction with FliM (55–57). Thus, GTP hydrolysis regulates the sequence of assembly for basal body components, apart from regulating the site for the biogenesis of the polar flagellum. The GTP hydrolysis by FlhF is regulated by YlxH in *B. subtilis* (29). The structure of FlhF complexed to the N-terminal activator helix of FlhG shows that the GTPase domain of FlhF interacts with the N-terminus activator helix. Furthermore, a glutamine present in the DQAXXLR motif is responsible for stimulating the activity of FlhF. Interestingly, FleN, the homolog of FlhG or YlxH in Pseudomonads, lacks the N-terminal DQAXXLR motif, indicating a distinct mechanism in this genus (1, 43).

FlhF is highly conserved among *Pseudomonas* species such as *P. putida, P. fluorescens, P. marginalis,* and *P. syringae*, with sequence identity ranging from about 60% to 70%. Similarly, FliG from *P. aeruginosa* shares above 85% sequence identity in these species. This high level of conservation strongly suggests that the function of different domains is likely to be conserved across these species. Consequently, the role of each domain in flagellar assembly and polar localization may be similar, reflecting evolutionary conservation of key molecular mechanisms governing flagellation.

## MATERIALS AND METHODS

### Strains and plasmids

The strains and plasmids used in the study are listed in Table 1. To grow *P. aeruginosa* and *E. coli* strains, LB medium was used.

**TABLE 1:** Strains and plasmids used in the study

Strain or plasmid	Description	Source
Strains		
BTH101	Adenylate cyclase-deficient (*cya) E. coli* reporter strains, used for bacterial two-hybrid assay	Euromedex
PAO1	Wild-type strain	ATCC
*ΔflhF*	Transposon insertional mutant	Manoil lab
*ΔfliG*	Transposon insertional mutant	Manoil lab
Plasmids		
pHERD20T	*Escherichia-Pseudomonas* shuttle vector, L-arabinose inducible pBAD promoter, Amp^r^	Gift from Prof. DN Rao, IISc
pHERD20T-*flhF_FL_*	Full-length *flhF* cloned in L-arabinose inducible vector pHERD20T, Amp^r^, used for complementation	This study
pHERD20T-*flhF_BN_*	*flhF_BN_* cloned in L-arabinose inducible vector pHERD20T, Amp^r^, used for complementation	This study
pHERD20T-*flhF_NG_*	*flhF_NG_* cloned in L-arabinose inducible vector pHERD20T, Amp^r^, used for complementation	This study
pHERD20T-*flhF_G_*	*flhF_G_* cloned in L-arabinose inducible vector pHERD20T, Amp^r^, used for complementation	This study
pHERD20T-*flhF_FL_-GFP*	Full-length *flhF* cloned in-frame with GFP in L-arabinose inducible vector pHERD20T, Amp^r^, used for localization studies	This study
pHERD20T-*flhF_BN_-GFP*	*flhF_BN_* cloned in-frame with GFP in L-arabinose inducible vector pHERD20T, Amp^r^, used for localization studies	This study
pHERD20T-*flhF_NG_-GFP*	*flhF_NG_* cloned in-frame with GFP in L-arabinose inducible vector pHERD20T, Amp^r^, used for localization studies	This study
pHERD20T-*flhF_G_-GFP*	*flhF_G_* cloned in-frame with GFP in L-arabinose inducible vector pHERD20T, Amp^r^, used for localization studies	This study
pGEX-6P-1-*flhF*	Vector for over-expression of N-terminal GST-tagged FlhF, Amp^r^	This study
pet14b-SUMO-modified-*fliG*	Vector for over-expression of N-terminal SUMO-His-tag FliG, Amp^r^	This study
pUT18C	Vector encoding the multiple cloning site at the C-terminal end of the T18 fragment of CyaA, used for bacterial two-hybrid assay, Amp^r^	Euromedex
pKT25	Vector encoding the multiple cloning site to the C-terminal end of the T25 fragment of CyaA, used for bacterial two-hybrid assay, Kan^r^	Euromedex
pUT18	Vector encoding the multiple cloning site to the N-terminal end of the T18 fragment of CyaA, used for bacterial two-hybrid assay, Amp^r^	Euromedex
pKNT25	Vector encoding the multiple cloning site to the N-terminal end of the T25 fragment of CyaA, used for bacterial two-hybrid assay, Kan^r^	Euromedex
pUT18C-*zip*	Leucine zipper of GCN4 fused to the C-terminal of T18 fragment, used as positive control in bacterial two-hybrid assay	Euromedex
pKT25-*zip*	Leucine zipper of GCN4 fused to the C-terminal of T25 fragment, used as a positive control in bacterial two-hybrid assay	Euromedex
pKNT25 *flhF*	Vector encoding chimeric FlhF fused to the N-terminal end of the T25 fragment of CyaA, used for bacterial two-hybrid assay, Amp^r^	This study
pUT18C *fliG*	Vector encoding chimeric FliG fused to the C-terminal end of the T18 fragment of CyaA, used for bacterial two-hybrid assay, Amp^r^	This study
pUT18 *flhF*	Vector encoding chimeric FlhF fused to the N-terminal end of the T18 fragment of CyaA, used for bacterial two-hybrid assay, Amp^r^	This study
pKT25 *flhF*	Vector encoding chimeric FlhF fused to the C-terminal end of the T25 fragment of CyaA, used for bacterial two-hybrid assay, Kan^r^	This study
pKT25 *fliG*	Vector encoding chimeric FliG fused to the C-terminal end of the T25 fragment of CyaA, used for bacterial two-hybrid assay, Kan^r^	This study

### Cloning of flhF and its constructs in pHERD20T for complementation

The nucleotides spanning the *flhF_FL_*, *flhF_BN_*, *flhF_NG_*, and *flhF_G_* were PCR amplified using the primers engineered with EcoRI and SmaI restriction sites (Table 2). The shuttle vector pHERD20T was digested with the same set of enzymes, and ligation was performed at 16°C. The ligated product was transformed into *E. coli* DH5α cells. The clones were confirmed through double digestion and further sequenced.

**TABLE 2:** Primers used in the study

Primer	Sequence (5′ to 3′)
EcoRI-*flhF*-forw	ATCGACGAATTCATGCAAGTCAAACGCTTCTTC
SmaI-*flhF*-rev	TCATCTCCCGGGTCAGCCGGCACG
SmaI-*BN*-rev	TATTCACCCGGGTCAGCCGCCCGCGTC
EcoRI-*NG*-forw	ATCGCTGAATTCATGCAGACCCTCGAGGCA
SalI-*GFP*-forw	ATCGATGTCGACGGAGAAGAAAAAATGAGTAAAGGA
HindIII-*GFP*-rev	ATCGTAAAGCTTTTTGTATAGTTCATCCATACCGTGTGT
SalI-*flhF*-forw	CGATGAGCTCGCAAGTCAAACGCTTCTTCGCC
SacI-*flhF*-rev	GACTGAGCTCTCAGCCGGCACGCCGCGCC
SalI-*NG*-forw	ATCGATGTCGACACAGACCCTCGAGGCAATGCGT
SacI-*BN*rev	TATTCAGAGCTCTCAGCCGCCCGCGTC
*Sal*I-*G*forw	ATCGATGTCGACAGAGCAGGACCCGCTCGAC
XbaI*-flhF*-forw	TATAGCTCTAGAGATGCAAGTCAAACGCTTCTTCGCC
SmaI-*flhF*-rev	TAGATACCCGGGGGCCGGCACGCCGCGCCGGTTG
BamHI-*fliG*-forw	ATCGATGGATCCAATGAGTGAGAATCGTCTCGCC
KpnI-*fliG*-rev	AGCTGAGGTACCTCAGATCATCTCCTCGCC
*BamH*I*-fliG*-forw	ATCGATGGATCCGCATGAGTGAGAATCGTCTCGCC
NdeI-*fliG*-forw	CGCCATATGATGAGTGAGAATCGTCTCGCC
BamHI-*fliG*-rev	GAAGGATCCTTACTCATCGGCGTTGATCCA

*flhF* knockout strains of *P. aeruginosa* were procured from Manoil lab (58). To prepare competent cells of *ΔflhF*, a glycerol stock of the same was streaked on an LB plate without any antibiotics. The next day, the primary culture was set up in 5 mL of LB using a single isolated colony. 500 µL of primary culture was used as an inoculum in 50 mL of LB to set up secondary culture, which was grown at 37°C at 180 rpm until the OD_600_ reached 0.4–0.5. The culture was pelleted at 2,146 × *g* for 10 minutes, and the pellet was resuspended in 50 mL of 300 mM saccharose solution. The cell suspension was spun at 1,200 × *g* for 10 minutes, and the pellet was again resuspended in 25 mL of saccharose solution, followed by spinning at 1,200 × *g* for 10 minutes. The final pellet collected after the third centrifugation was resuspended in 500 µL of saccharose, and aliquots of 80 µL were prepared and used immediately for electroporation.

For electroporation, 100 ng of either the pHERD20T: *flhF_FL_*, pHERD20T: *flhF_BN_*, pHERD20T: *flhF_NG_,* or pHERD20T: *flhF_G_* was added to 80 µL aliquots of the Δ*flhf P. aeruginosa* cells and incubated on ice for 30 minutes. The cells were then transferred to a pre-cooled electro cuvette, and the cuvette was placed in Gene Pulser X (BioRad). The voltage was applied at 2,500 Volts for 25 ms, and cells were immediately transferred into 2 mL of SOC (Super Optimal broth with Catabolite repression comprising of 0.5% yeast extract, 2% tryptone, 10 mM NaCl, 2.5 mM KCl, and 20 mM MgSO_4_). After autoclaving, 22 mM of glucose was added to the media and mixed well. The cells were grown at 37°C for 2 hours in a shaker incubator at 200 rpm. The cells were pelleted and resuspended in SOC media before plating them on 300 µg/mL carbenicillin-containing LB-Agar plates.

### Motility assay

The swimming motility was assessed using soft agar plates composed of LB medium with 0.3% agar. The plates were dried for 20 minutes before inoculation. The plates were poked with a single colony each of wild-type strain PAO1, Δ*flhF*, and Δ*flh*F complemented with *flhF_FL_, flhF_BN_, flhF_NG_,* and *flhF_G_* using sterile toothpicks in the presence of 0.25% arabinose wherever required, and the plates were incubated at 37°C for 16 hours. The diameter was measured for the spread of the bacterial cells from the point of inoculation. The diameter of each circle was measured from three directions, and the average was plotted as a bar graph in Microsoft Excel. The standard deviation was calculated using three independent biological replicates.

### Transmission electron microscopy

Prior to application of the bacterial culture, the carbon-coated grids CF300-CU (Electron Microscopy Sciences) were glow-discharged for 30 seconds. An aliquot of 4 µL of secondary culture of Δ*flhF,* complemented with either *flhF_FL_* or *flhF_BN_,* or *flhF_NG_,* or *flhF_G_* domains, was grown to an OD_600_ of 0.4–0.6, and then deposited on a charged carbon-coated copper grid and left to adhere for 5 minutes. The extra culture was blotted using Whatman’s filter paper. Grids were washed twice with distilled water, followed by negative staining using 2% phosphotungstic acid. Extra stain was blotted, and grids were dried before imaging. A minimum of 100 cells was counted in each experiment, and the experiment for each strain was repeated three times.

### Construction of GFP-tagged fusion protein

To determine the intracellular localization of FlhF, the *flhF* full-length (*flhF_FL_*) and various domain combinations, *flhF_BN_, flhF_NG_,* and *flhF*_G_ were PCR amplified using the primers mentioned in Table 2. The amplified and digested products were then cloned into an arabinose-inducible shuttle vector pHERD20T using the enzymes EcoRI and SmaI. The *GFP* was separately amplified from a pJN105-Hygro-GFP plasmid (Addgene) using the primers mentioned in Table 2 with SalI and HindIII restriction sites to generate pHERD20T-gene-GFP constructs. The colonies obtained were screened for the desired clone and sequenced. All the enzymes used were procured from New England Biolabs.

### Confocal microscopy

Confocal microscopy was performed to determine the intracellular localization of the GFP-tagged protein. The Δ*flhF* cells complemented with *gfp* fused *flhF* full-length (*flhF_FL_*) and various domain combinations, *flhF_BN_, flhF_NG_,* and *flhF*_G_ were cultured at 37°C to an OD_600_ of 0.4–0.5. To induce the expression of GFP-tagged proteins, 0.5% arabinose was added to the culture. Further cells were allowed to grow for 3 hours and kept at 4°C overnight before imaging. 1 mL of culture was centrifuged at 600 × *g* for 2 minutes. Cells were resuspended in 200 µL of phosphate-buffered saline (PBS) after giving a gentle wash. 100 µL of resuspended cells was placed on a glass slide sealed with a coverslip and observed at 60× magnification in a laser scanning microscope. Images were quantified using ImageJ software (59). The dot plot was created using GraphPad Prism version 8.4.3.

### Purification of full-length FlhF

To purify the full-length FlhF protein, the *flhF_FL_* was cloned into the pGEX-6P1 vector using primers (Table 2) containing EcoRI and NotI restriction sites, with a GST tag at the N-terminus. The plasmid (100 ng) was transformed into 100 µL BL21 (DE3) pLysS-competent cells. The cells were inoculated in 5 L of LB in the presence of ampicillin 100 µg/mL and chloramphenicol 34 µg/mL. When the culture reached the OD_600_ of 0.8, 0.5 mM isopropyl β-D-1-thiogalactopyranoside (IPTG) was added, and the culture was grown at 18°C to ensure expression of protein. The cells were pelleted, and lysis buffer (25 mM Tris pH 8, 500 mM NaCl, 10 mM MgCl_2_, 10% glycerol, 2 mM DTT, and 1 mM PMSF) was used to resuspend the pellet. To lyse the cells, the cell lysate was subjected to sonication using a 10-second pulse with an off cycle of 59 seconds. This cycle was repeated three times. Three such major cycles were used. The lysed cells were spun at 21,890 × *g*, and the supernatant was filtered using 0.45 micron filters. The supernatant was loaded onto a GST column containing 5 mL GST beads. The beads were immersed in the supernatant containing the recombinant protein and stirred at regular intervals. The beads were washed with 100 mL of 25 mM Tris pH 8, 300 mM NaCl, 10 mM MgCl_2_, 7% glycerol, and 2 mM DTT (low-salt buffer) to remove unbound protein. To remove non-specifically bound proteins, a high salt buffer containing 1 M NaCl, 25 mM Tris pH 8, 7% glycerol, 10 mM MgCl_2_, and 2 mM DTT was used for washing. Again, a 50 mL of low-salt buffer was used to wash the beads. To cleave the N-terminal GST tag, precision protease was added to the beads in 5 mL of low-salt buffer. Cleaved protein was eluted as 5 mL fractions in low-salt buffer. The elution fractions were run on a 12% SDS-PAGE gel to test the purity of the protein. The fractions corresponding to pure protein were pooled, concentrated using 10 kDa (Sartorius) concentrators, and stored at −80°C.

### Purification of FliG

The *fliG* cloned in the modified pET-14b with an N-terminal sumo-His tag was transformed into BL21 (DE3) cells. To set up a large volume of cultures, 2 L of LB containing 100 µg/mL ampicillin was inoculated with transformed colonies and grown until the OD_600_ reached 0.8 at 37°C and 180 rpm. 0.5 mM IPTG was added, and cultures were grown at 18°C to induce protein expression. After induction, the cells were pelleted and resuspended in lysis buffer containing 25 mM Tris, pH 8, 2% glycerol, 250 mM NaCl, 5 mM imidazole, and 1 mM EDTA. To lyse the cells, the cell lysate was subjected to sonication using a 10-second pulse with an off cycle of 59 seconds. This cycle was repeated three times. Three such major cycles were used. The lysed cells were spun at 21,890 × *g*, and the supernatant was filtered using 0.4 micron filters. The supernatant was loaded onto a 5 mL Ni-NTA HisTrap column (Cytiva). The protein was loaded on the column in binding buffer (25 mM Tris pH 8, 2% glycerol, 250 mM NaCl) and eluted using a step gradient of imidazole using elution buffer (25 mM Tris pH 8, 2% glycerol, 250 mM NaCl, 1M imidazole). Precision protease was added to the eluted fractions during dialysis in a buffer containing 25 mM Tris pH 8, 2% glycerol, and 250 mM NaCl to cleave the tag. To separate the untagged protein from the SUMO-His tag, a second round of Ni-NTA chromatography was performed. The protein was subsequently purified through size-exclusion chromatography using Superdex 200 10/300 Gl (Cytiva) for further purification.

### Bacterial two-hybrid assay

To investigate the interaction between FlhF and FliG, the genes encoding *flhF* and *fliG* were amplified through colony PCR from the PAO1 strain of *P. aeruginosa* and cloned into the pUT18, pUT18c, pKNT25, and pKT25 vectors that express complementary fragments of bacterial adenylate cyclase CyaA (Euromedex). BTH101 cells were co-transformed with sets of compatible plasmids and plated on an indicator plate containing 0.5 mM IPTG and 40 µg/mL of X-gal (5-bromo-4-chloro-3-indolyl-beta-D-galactopyranoside). The plates were incubated at 30°C for 2–3 days. The colonies from each selection plate were cultured in LB media at 30°C containing 50 µg/mL kanamycin and 100 µg/mL ampicillin. The cultures were induced with 0.5 mM IPTG. Spotting was performed on MacConkey plates containing 1% maltose and LB plates supplemented with IPTG and X-gal.

To perform the β-galactosidase assay, overnight-grown cultures were adjusted to an OD_600_ of 0.5. Cells were permeabilized using monosodium phosphate buffer (20 mM potassium chloride, 2 mM magnesium sulfate, 2 mM Cetyltrimethylammonium bromide, 1 mM sodium deoxycholate, and 77 mM beta-mercaptoethanol) for 20 min at room temperature. 600 µL of 6.5 mM ortho-nitrophenyl β-galactoside prepared in solubilization buffer (20 mM potassium chloride, 2 mM magnesium sulfate, 2 mM CTAB or cetyltrimethylammonium bromide, 1 mM sodium deoxycholate) was added to each tube and incubated for at least 30 min. To stop the reaction, 700 µL of 1 M sodium carbonate was added to each tube as the color developed. The tubes were spun at 9,659 × *g* for 5 min to separate the supernatant from the debris. The supernatant was diluted to a ratio of 1:10 using the solubilization buffer, and absorbance was recorded at 420 nm and 550 nm.

### Dynamic light scattering

To perform size distribution analysis, 0.01 mM each of FlhF and FliG was used. The proteins were diluted using a buffer containing 25 mM Tris, pH 8, and 10 mM MgCl_2_. The protein mix was spun at 15,093 × *g* rpm for an hour before recording the readings using a Malvern instrument. To prepare the mix of proteins in the presence of nucleotides, 10-fold of either GMPPNP or GDP was added to FlhF, and the mix was incubated on ice for 40 minutes before adding FliG. After FliG was added, the mix was incubated for an additional 45 minutes on ice. 60 readings were recorded for each run using Zetasizer software. The readings for individual proteins were also recorded as controls. The intensity distribution was plotted in MS Excel.

### Flagellin isolation

To isolate and visualize flagellin, 200 mL of culture was set up for PAO1, *ΔflhF*, *ΔflhF* complemented with *flhF_FL_, flhF_BN_, flhF_NG_*, and *flhF_G_* in the presence of 0.25% arabinose and grown to an OD_600_ of 0.6. The cultures were pelleted down at 3,000 × *g* for 20 minutes. The cell pellets were resuspended in 15 mL PBS and vortexed for 20 minutes to shear off the flagella. The vortexed cells were spun at 10,000 × *g* for 30 minutes. The supernatant was collected and centrifuged at 100,000 × *g* for 2 hours to obtain a pellet of flagellin. The pellet was resuspended in 500 µL autoclaved water, and 20 µL of each sample was mixed with 6× SDS loading buffer and run on a 10% SDS-PAGE gel. The gel was stained with Coomassie Brilliant Blue.

### Western blot analysis

To detect the expression of FlhF and its constructs, Western Blot analysis was performed. 50 mL of culture was set up each for *ΔflhF* complemented with *flhF_FL_-gfp*, *flhF_BN_-gfp*, *flhF_NG_-gfp*, *flhF_G_-gfp*, and *gfp* alone, grown to an OD_600_ of 0.6 in the presence of 0.5% arabinose. The cultures were incubated at 4°C overnight. The next day, the cultures were pelleted at 5,000 × *g*. The pellets were resuspended in 30 mL lysis buffer containing 25 mM Tris, pH 8, 500 mM NaCl, 7% glycerol, and 10 mM magnesium chloride supplemented with a protease inhibitor. The cells were lysed by 5 cycles of sonication. 40 µL of lysate was added with 6× SDS-loading buffer and run on 10% SDS-PAGE gels. The proteins were transferred overnight onto a polyvinylidene fluoride membrane activated with methanol at 30 volts. The membrane was blocked with 5% skim milk for 1 hour at room temperature, followed by washing in PBS with 0.1% Tween (PBST). The membrane was incubated with anti-GFP (1:3,000) antibody overnight at 4°C. The membrane was washed thrice with PBST, followed by incubation with HRP-linked rabbit anti-mouse IgG polyclonal antibodies (1:5,000) for 2 hours and visualized using chemiluminescence.

### Statistical analysis

The data presented in the bar charts and dot plots are mean values ± SD, or as indicated in the legend provided beneath the respective figure. Statistical significance was analyzed using an unpaired t-test with GraphPad Prism software v.10.0.3 (GraphPad Software, La Jolla, CA, USA).

## Data Availability

The data supporting the findings in the study were collected at the Regional Centre for Biotechnology, Faridabad, and are available in the article and its supplemental material.

## SUPPLEMENTAL MATERIAL

Supplemental figures jb.00332-25-s0001.pdf Figures S1 to S3.

ASM does not own the copyrights to Supplemental Material that may be linked to, or accessed through, an article. The authors have granted ASM a non-exclusive, world-wide license to publish the Supplemental Material files. Please contact the corresponding author directly for reuse.
