# Supplementary material for: Role of FlhF and its domains in the assembly of a polar flagellum in P. aeruginosa
Source: J Bacteriol. 2025 Nov 24;207(12):e00332-25. doi: 10.1128/jb.00332-25 (PMC12713375; doi:10.1128/jb.00332-25)
Supplement: Supplemental figures — Figures S1 to S3. [file jb.00332-25-s0001.pdf]

Fig. S1

A- Wild type
B- ΔflhF
C- PAO1 + flhF
D- PAO1 + flhF BN
E- PAO1 + flhF NG
F- PAO1 + flhF G

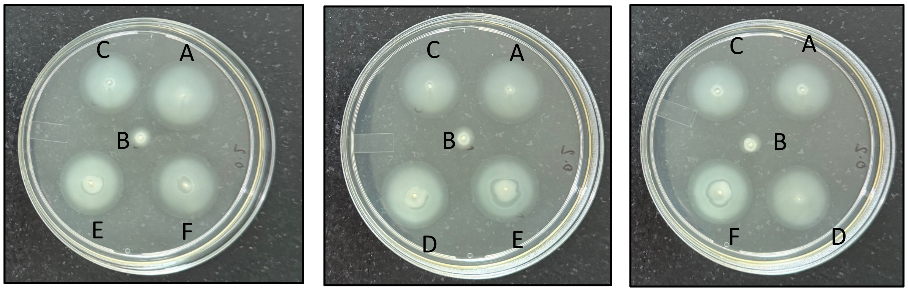

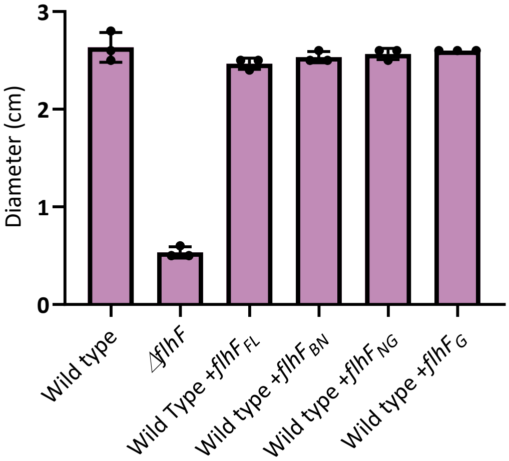

Fig S1. Image of soft agar plate depicting swimming motility of the wild type PAO1, *flhF* knockout (*ΔflhF*), wild type complemented with either $flhF_{FL}$, $flhF_{BN}$, $flhF_{NG}$ or $flhF_{G}$ domains. The bar graph represents the average diameter from the three independent experiments.

Fig. S2

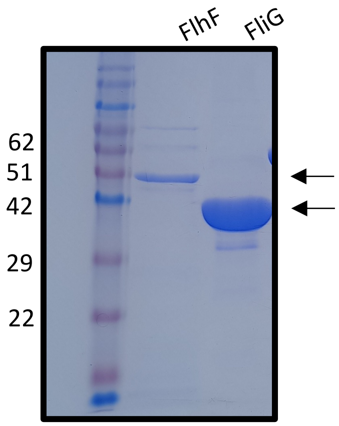

Fig S2. 12% SDS-PAGE gel showing the purity of the purified FlhF and FliG corresponding to their respective molecular weights.

Fig. S3.

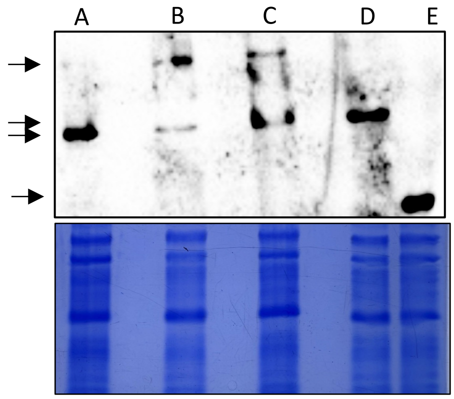

A- $FlhF_{BN}$-GFP
B- $FlhF_{FL}$-GFP
C- $FlhF_{NG}$-GFP
D- $FlhF_{G}$-GFP
E- GFP alone

Fig S3. Cell lysates from different GFP-complementation constructs were separated on 10% SDS-PAGE gel and probed against GFP to visualize the protein in western blotting. The bands corresponding to $FlhF_{BN}$ (50kDa), $FlhF_{FL}$ (75kDa), $FlhF_{NG}$ (61kDa), $FlhF_{G}$ (51kDa), and GFP control (27kDa) are marked with black arrows.
